# (2*Z*)-Methyl 2-(2-amino-1,3-thia­zol-4-yl)-2-(methoxy­imino)ethano­ate

**DOI:** 10.1107/S1600536809019643

**Published:** 2009-06-06

**Authors:** Shahzad Sharif, M. Nawaz Tahir, Islam Ullah Khan, Manan Ayub Salariya, Sarfraz Ahmad

**Affiliations:** a Department of Chemistry, Government College University, Lahore, Pakistan; b Department of Physics, University of Sargodha, Sargodha, Pakistan; c Pharmagen Ltd, Lahore 54000, Pakistan

## Abstract

In the title compound, C_7_H_9_N_3_O_3_S, the planes of the 2-amino-1,3-thia­zol-4-yl and the methyl ester groups are oriented at a dihedral angle of 67.06 (7)°. In the crystal, inversion dimers linked by pairs of N—H⋯N hydrogen bonds occur, forming *R*_2_^2^(8) ring motifs. The dimers are inter­linked by N—H⋯O hydrogen bonds, resulting in sheets propagating in the *ac* plane.

## Related literature

For a related structure, see: Laurent *et al.* (1981 ▶). For background to the use of the title compound in organic synthesis, see: Khanna *et al.* (1999 ▶). For graph-set notation, see: Bernstein *et al.* (1995 ▶);
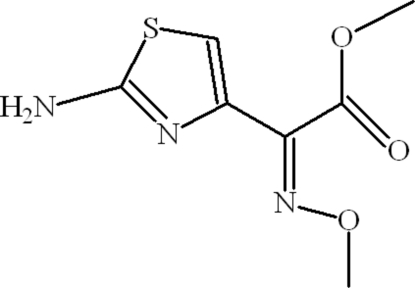


## Experimental

### 

#### Crystal data

C_7_H_9_N_3_O_3_S
*M* *_r_* = 215.23
Monoclinic, 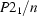

*a* = 7.8096 (4) Å
*b* = 8.1994 (5) Å
*c* = 15.6247 (9) Å
β = 92.936 (2)°
*V* = 999.20 (10) Å^3^
*Z* = 4
Mo *K*α radiation
μ = 0.31 mm^−1^
*T* = 296 K
0.25 × 0.20 × 0.18 mm

#### Data collection

Bruker Kappa APEXII CCD diffractometer
Absorption correction: multi-scan (*SADABS*; Bruker, 2005 ▶) *T*_min_ = 0.931, *T*_max_ = 0.945
9949 measured reflections
2295 independent reflections
1696 reflections with *I* > 2σ(*I*)
*R*_int_ = 0.028

#### Refinement

*R*[*F* ^2^ > 2σ(*F* ^2^)] = 0.040
*wR*(*F* ^2^) = 0.120
*S* = 1.03
2295 reflections
135 parameters
H atoms treated by a mixture of independent and constrained refinement
Δρ_max_ = 0.26 e Å^−3^
Δρ_min_ = −0.16 e Å^−3^

### 

Data collection: *APEX2* (Bruker, 2007 ▶); cell refinement: *SAINT* (Bruker, 2007 ▶); data reduction: *SAINT*; program(s) used to solve structure: *SHELXS97* (Sheldrick, 2008 ▶); program(s) used to refine structure: *SHELXL97* (Sheldrick, 2008 ▶); molecular graphics: *ORTEP-3* (Farrugia, 1997 ▶) and *PLATON* (Spek, 2009 ▶); software used to prepare material for publication: *WinGX* (Farrugia, 1999 ▶) and *PLATON*.

## Supplementary Material

Crystal structure: contains datablocks global, I. DOI: 10.1107/S1600536809019643/hb2983sup1.cif

Structure factors: contains datablocks I. DOI: 10.1107/S1600536809019643/hb2983Isup2.hkl

Additional supplementary materials:  crystallographic information; 3D view; checkCIF report

## Figures and Tables

**Table 1:** Hydrogen-bond geometry (Å, °)

*D*—H⋯*A*	*D*—H	H⋯*A*	*D*⋯*A*	*D*—H⋯*A*
N2—H2*A*⋯O1^i^	0.83 (3)	2.28 (2)	3.058 (2)	156 (2)
N2—H2*B*⋯N1^ii^	0.84 (3)	2.20 (3)	3.024 (2)	166 (3)

Symmetry codes: (i) 
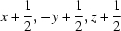
; (ii) 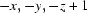.

**Fig. 1.**
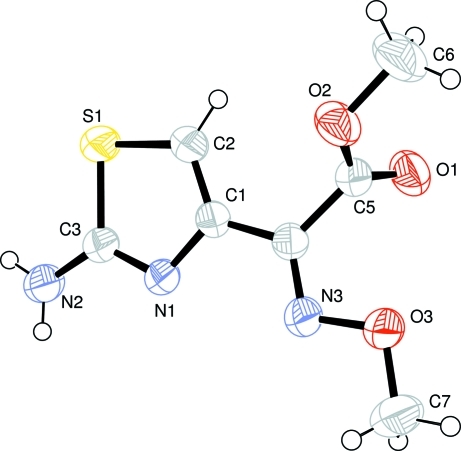
View of (I) with displacement ellipsoids drawn at the 50% probability level. H-atoms are shown by small spheres of arbitrary radius.

**Fig. 2.**
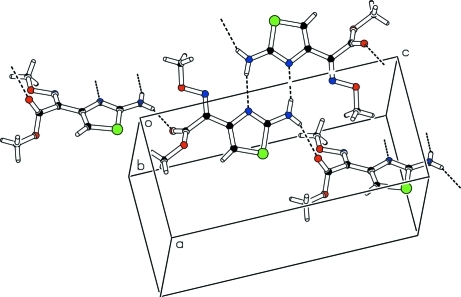
The partial packing of (I) which shows that molecules form dimers and the dimers are interlinked forming two dimensional polymeric sheets.

## supplementary crystallographic information

### Comment

2-Mercapto-benzothiazolyl-(*Z*)-2-(2-aminothiazol-4-yl)-2-methoxyimino acetate (MAEM) is a standard acylating agent for the preparation of cephalosporins (Khanna *et al.*, 1999). The title compound (I), (Fig 1), is prepared as an intermediate for derivitaziation.

The crystal structure of (II) Ethyl 2-amino-α-(*E*-methoxyimino)-4-thiazoleacetate (Laurent *et al.*, 1981) has been published. (I) differs from (II) due to the methoxy group attached with carbonyl instead of ethoxy moiety.

The title compound is dimerized due to the intermolecular H-bonding of N—H···N type forming *R*_2_^2^(8) ring motifs (Bernstein *et al.*, 1995). The dimers are further linked with each other through the intermolecular H-bonding of N—H···O type (Table 1), (Fig. 2). The five membered ring along with NH_2_ A (C1/C2/S1/C3/N1/N2), methyl ester group B (O1/C5/O2/C6) and the group C (C4/N3/O3/C7) are planar. The dihedral angles between A/B, A/C and B/C have values of 67.06 (7), 9.21 (16) and 71.67 (11)°, respectively.

### Experimental

2-Mercapto-benzothiazolyl-(*Z*)-2-(2-aminothiazol-4-yl)-2-methoxyimino acetate (0.2 g, 1.4 mmol) was dissolved in methanol (5 ml) and stirred for 1 h at 303 K.  Yellow prisms of (I) were obtained through slow evaporation after five days.

### Refinement

The coordinates of H-atoms of NH_2_ group were refined. Other H atoms were positioned geometrically, with C—H = 0.93 and 0.96 Å for aryl and methyl H, respectively and constrained to ride on their parent atoms, with *U*_iso_(H) = *xU*_eq_(C, N), where *x* = 1.5 for methyl and 1.2 for other H atoms.

### Crystal data

**Table:**

C_7_H_9_N_3_O_3_S	*F*(000) = 448
*M**_r_* = 215.23	*D*_x_ = 1.431 Mg m^−^^3^
Monoclinic, *P*2_1_/*n*	Mo *K*α radiation, λ = 0.71073 Å
Hall symbol: -P 2yn	Cell parameters from 2295 reflections
*a* = 7.8096 (4) Å	θ = 2.6–27.5°
*b* = 8.1994 (5) Å	µ = 0.31 mm^−^^1^
*c* = 15.6247 (9) Å	*T* = 296 K
β = 92.936 (2)°	Prism, yellow
*V* = 999.20 (10) Å^3^	0.25 × 0.20 × 0.18 mm
*Z* = 4	

### Data collection

**Table:**

Bruker Kappa APEXII CCD diffractometer	2295 independent reflections
Radiation source: fine-focus sealed tube	1696 reflections with *I* > 2σ(*I*)
graphite	*R*_int_ = 0.028
Detector resolution: 7.50 pixels mm^-1^	θ_max_ = 27.5°, θ_min_ = 2.6°
ω scans	*h* = −10→10
Absorption correction: multi-scan (*SADABS*; Bruker, 2005)	*k* = −8→10
*T*_min_ = 0.931, *T*_max_ = 0.945	*l* = −20→20
9949 measured reflections	

### Refinement

**Table:**

Refinement on *F*^2^	Primary atom site location: structure-invariant direct methods
Least-squares matrix: full	Secondary atom site location: difference Fourier map
*R*[*F*^2^ > 2σ(*F*^2^)] = 0.040	Hydrogen site location: inferred from neighbouring sites
*wR*(*F*^2^) = 0.120	H atoms treated by a mixture of independent and constrained refinement
*S* = 1.03	*w* = 1/[σ^2^(*F*_o_^2^) + (0.0635*P*)^2^ + 0.2265*P*] where *P* = (*F*_o_^2^ + 2*F*_c_^2^)/3
2295 reflections	(Δ/σ)_max_ < 0.001
135 parameters	Δρ_max_ = 0.26 e Å^−^^3^
0 restraints	Δρ_min_ = −0.16 e Å^−^^3^

### Special details

**Table:**

Geometry. Bond distances, angles *etc*. have been calculated using the rounded fractional coordinates. All su's are estimated from the variances of the (full) variance-covariance matrix. The cell e.s.d.'s are taken into account in the estimation of distances, angles and torsion angles
Refinement. Refinement of *F*^2^ against ALL reflections. The weighted *R*-factor *wR* and goodness of fit *S* are based on *F*^2^, conventional *R*-factors *R* are based on *F*, with *F* set to zero for negative *F*^2^. The threshold expression of *F*^2^ > σ(*F*^2^) is used only for calculating *R*-factors(gt) *etc*. and is not relevant to the choice of reflections for refinement. *R*-factors based on *F*^2^ are statistically about twice as large as those based on *F*, and *R*- factors based on ALL data will be even larger.

### Fractional atomic coordinates and isotropic or equivalent isotropic displacement parameters (Å^2^)

**Table:**

	*x*	*y*	*z*	*U*_iso_*/*U*_eq_	
S1	0.41857 (6)	0.20584 (8)	0.43460 (3)	0.0599 (2)	
O1	0.04763 (18)	0.26609 (19)	0.13478 (9)	0.0637 (5)	
O2	0.25926 (17)	0.08248 (18)	0.14230 (9)	0.0599 (5)	
O3	−0.14991 (17)	−0.0224 (2)	0.17911 (8)	0.0606 (5)	
N1	0.11714 (18)	0.0792 (2)	0.41045 (9)	0.0475 (5)	
N2	0.2110 (2)	0.0993 (3)	0.55484 (11)	0.0719 (8)	
N3	−0.06554 (18)	−0.0011 (2)	0.26002 (9)	0.0482 (5)	
C1	0.1775 (2)	0.1176 (2)	0.33140 (11)	0.0419 (5)	
C2	0.3342 (2)	0.1859 (3)	0.33191 (12)	0.0519 (6)	
C3	0.2315 (2)	0.1199 (3)	0.47114 (12)	0.0483 (6)	
C4	0.0712 (2)	0.0836 (2)	0.25360 (11)	0.0412 (5)	
C5	0.1219 (2)	0.1552 (2)	0.17005 (11)	0.0452 (6)	
C6	0.3165 (3)	0.1371 (4)	0.05994 (15)	0.0826 (10)	
C7	−0.3004 (3)	−0.1162 (4)	0.18950 (16)	0.0913 (12)	
H2	0.38901	0.21784	0.28319	0.0622*	
H2A	0.288 (3)	0.129 (3)	0.5901 (17)	0.0863*	
H2B	0.124 (4)	0.053 (3)	0.5734 (17)	0.0863*	
H6A	0.24081	0.09504	0.01485	0.1240*	
H6B	0.43070	0.09816	0.05256	0.1240*	
H6C	0.31566	0.25411	0.05803	0.1240*	
H7A	−0.27198	−0.21154	0.22295	0.1370*	
H7B	−0.34834	−0.14852	0.13428	0.1370*	
H7C	−0.38262	−0.05196	0.21829	0.1370*	

### Atomic displacement parameters (Å^2^)

**Table:**

	*U*^11^	*U*^22^	*U*^33^	*U*^12^	*U*^13^	*U*^23^
S1	0.0470 (3)	0.0858 (4)	0.0465 (3)	−0.0188 (3)	−0.0024 (2)	0.0058 (3)
O1	0.0605 (9)	0.0762 (10)	0.0548 (9)	0.0119 (8)	0.0062 (7)	0.0219 (8)
O2	0.0583 (8)	0.0742 (10)	0.0486 (8)	0.0134 (7)	0.0177 (6)	0.0089 (7)
O3	0.0514 (7)	0.0897 (11)	0.0403 (7)	−0.0193 (7)	−0.0009 (5)	0.0000 (7)
N1	0.0366 (7)	0.0672 (10)	0.0385 (8)	−0.0026 (7)	0.0012 (6)	0.0057 (7)
N2	0.0506 (10)	0.1262 (19)	0.0386 (9)	−0.0213 (11)	−0.0016 (7)	0.0093 (10)
N3	0.0444 (8)	0.0626 (10)	0.0374 (8)	−0.0034 (7)	0.0015 (6)	−0.0006 (7)
C1	0.0397 (8)	0.0468 (10)	0.0392 (9)	0.0019 (7)	0.0019 (7)	0.0054 (8)
C2	0.0480 (10)	0.0666 (12)	0.0410 (9)	−0.0114 (9)	0.0023 (8)	0.0065 (9)
C3	0.0383 (8)	0.0634 (12)	0.0431 (10)	−0.0017 (8)	0.0019 (7)	0.0053 (9)
C4	0.0377 (8)	0.0475 (10)	0.0385 (9)	0.0033 (7)	0.0039 (6)	0.0016 (8)
C5	0.0415 (9)	0.0546 (11)	0.0394 (9)	−0.0019 (8)	0.0010 (7)	0.0015 (8)
C6	0.0799 (16)	0.112 (2)	0.0588 (14)	0.0138 (15)	0.0323 (12)	0.0181 (14)
C7	0.0640 (14)	0.142 (3)	0.0674 (15)	−0.0480 (16)	−0.0010 (12)	−0.0012 (16)

### Geometric parameters (Å, °)

**Table:**

S1—C2	1.7109 (19)	N2—H2B	0.84 (3)
S1—C3	1.7442 (18)	C1—C2	1.346 (2)
O1—C5	1.198 (2)	C1—C4	1.463 (2)
O2—C5	1.320 (2)	C4—C5	1.503 (2)
O2—C6	1.454 (3)	C2—H2	0.9300
O3—N3	1.4062 (19)	C6—H6A	0.9600
O3—C7	1.421 (3)	C6—H6B	0.9600
N1—C1	1.381 (2)	C6—H6C	0.9600
N1—C3	1.312 (2)	C7—H7A	0.9600
N2—C3	1.336 (3)	C7—H7B	0.9600
N3—C4	1.282 (2)	C7—H7C	0.9600
N2—H2A	0.83 (3)		
			
C2—S1—C3	88.83 (9)	O1—C5—O2	125.06 (16)
C5—O2—C6	116.32 (17)	O1—C5—C4	123.60 (15)
N3—O3—C7	108.47 (15)	O2—C5—C4	111.32 (14)
C1—N1—C3	109.73 (15)	S1—C2—H2	125.00
O3—N3—C4	110.53 (14)	C1—C2—H2	125.00
H2A—N2—H2B	118 (3)	O2—C6—H6A	109.00
C3—N2—H2B	122.2 (18)	O2—C6—H6B	109.00
C3—N2—H2A	119.5 (17)	O2—C6—H6C	109.00
C2—C1—C4	124.15 (16)	H6A—C6—H6B	109.00
N1—C1—C4	119.64 (14)	H6A—C6—H6C	109.00
N1—C1—C2	116.21 (16)	H6B—C6—H6C	109.00
S1—C2—C1	110.60 (14)	O3—C7—H7A	109.00
S1—C3—N1	114.63 (14)	O3—C7—H7B	109.00
S1—C3—N2	121.05 (14)	O3—C7—H7C	109.00
N1—C3—N2	124.33 (17)	H7A—C7—H7B	109.00
C1—C4—C5	118.93 (14)	H7A—C7—H7C	109.00
N3—C4—C1	118.53 (15)	H7B—C7—H7C	109.00
N3—C4—C5	122.49 (15)		
			
C3—S1—C2—C1	−0.42 (17)	O3—N3—C4—C5	3.3 (2)
C2—S1—C3—N1	0.46 (18)	N1—C1—C2—S1	0.3 (2)
C2—S1—C3—N2	−179.5 (2)	C4—C1—C2—S1	−179.86 (13)
C6—O2—C5—O1	−3.7 (3)	N1—C1—C4—N3	−9.3 (2)
C6—O2—C5—C4	177.57 (17)	N1—C1—C4—C5	168.00 (15)
C7—O3—N3—C4	−179.82 (18)	C2—C1—C4—N3	170.87 (19)
C3—N1—C1—C2	0.0 (2)	C2—C1—C4—C5	−11.8 (3)
C3—N1—C1—C4	−179.81 (17)	N3—C4—C5—O1	70.4 (2)
C1—N1—C3—S1	−0.4 (2)	N3—C4—C5—O2	−110.87 (18)
C1—N1—C3—N2	179.6 (2)	C1—C4—C5—O1	−106.8 (2)
O3—N3—C4—C1	−179.50 (14)	C1—C4—C5—O2	71.92 (19)

### Hydrogen-bond geometry (Å, °)

**Table:**

*D*—H···*A*	*D*—H	H···*A*	*D*···*A*	*D*—H···*A*
N2—H2A···O1^i^	0.83 (3)	2.28 (2)	3.058 (2)	156 (2)
N2—H2B···N1^ii^	0.84 (3)	2.20 (3)	3.024 (2)	166 (3)

Symmetry codes: (i) *x*+1/2, −*y*+1/2, *z*+1/2; (ii) −*x*, −*y*, −*z*+1.
